# Portuguese Adaptation and Psychometric Validation of the Brief Scale of Mood Regulation Through Music (B-MMR) with Students

**DOI:** 10.3390/bs16050761

**Published:** 2026-05-13

**Authors:** Ana Isabel Pereira, David Tomé-Lourido, Miguel-Ángel Hermida

**Affiliations:** 1 Centre for Music Studies (CESEM), School of Social Sciences and Humanities (NOVA FCSH), NOVA University of Lisbon, 1069-061 Lisbon, Portugal; 2 Facultad de Ciencias de la Salud, Universidad Internacional de La Rioja, 26006 Logroño, Spain; 3 Faculty of Education, Universidade da Coruña, 15001 Coruña, Spain; miguel.lopez.hermida@udc.es

**Keywords:** mood regulation, psychometric validation, music listening, cultural adaptation, emotional regulation

## Abstract

Music plays a significant role in mood regulation, influencing emotional states, reducing stress, and enhancing overall well-being. However, validated instruments to assess the use of music as a mood-regulation strategy are limited. This study aimed to adapt and validate the Brief Music in Mood Regulation Scale (B-MMR), a 21-item self-report measure, for use in Portuguese. Data were collected from 493 Portuguese students in an online survey. Participants completed the B-MMR alongside the Basic Emotions Questionnaire. Confirmatory factor analyses, reliability assessments, measurement invariance testing, and external validity analyses were conducted to evaluate the scale’s psychometric properties. Confirmatory factor analysis supported the original seven-factor structure, with three items per factor, with acceptable global fit indices. The Portuguese version showed satisfactory reliability. Invariance analyses revealed equivalence of the factor structure, loadings, and intercepts across sex groups, supporting meaningful comparisons of scores between men and women. Correlations between B-MMR dimensions and basic emotions were small to moderate and are interpreted as preliminary and indirect indicators of external validity, as they index emotional experiences rather than regulatory processes. Findings suggest that the Portuguese B-MMR is a promising tool for research on music-based mood regulation in student populations. A comprehensive validation across more diverse and clinical samples, using established emotion regulation measures, is needed before clinical or applied use.

## 1. Introduction

### 1.1. Music Listening and Mood-Regulation in Daily Life

Music listening is one of the most used everyday activities and plays a central role in how people manage their emotions and moods (Krause et al., 2015, 2021a). Individuals of different ages deliberately use music to enhance positive affect, cope with stress, regulate arousal and support self-reflection and social connectedness, making it a key resource for psychosocial development (Baltazar et al., 2019; Cascarino et al., 2021; Elvers et al., 2018; Krause et al., 2021b; Lonsdale & North, 2011; Schäfer et al., 2020). Within the broader literature on emotion and mood regulation, this can be understood as a domain-specific form of self-regulation: people strategically select and engage with music to influence their internal states in ways that complement more general regulatory strategies such as distraction, reappraisal or emotional expression (Brattico et al., 2011; Hargreaves & Lamont, 2017; Juslin, 2013; S. Saarikallio, 2007; S. H. Saarikallio et al., 2020; Schellenberg & Mankarious, 2012). For the adolescent age group in particular, emotional regulation emerges as a central motive for music listening (Kahn et al., 2025; Krause et al., 2015; S. Saarikallio et al., 2014; S. H. Saarikallio et al., 2020), reinforcing the relevance of instruments that assess music-based mood regulation strategies.

Self-regulation is the ability to regulate emotions and cognitive processes that shape people’s circumstances and their relationships with the environment (Pas et al., 2021). It encompasses aspects ranging from memory to work capacity, all of which directly affect executive functions and make individuals more adaptable to the situations they encounter (Hofmann et al., 2012). These processes can be described along four dimensions—cognitive, affective, motivational, behavioural, and contextual—within which music can serve as a flexible tool for stabilising and modulating emotional experience. The affective-motivational dimension concerns the awareness and control of thoughts and emotions, as well as their self-regulation (Harley et al., 2019; Pekrun, 2017). In this regard, the diversity of musical styles offers multiple avenues for seeking stability and emotional self-control, though effects vary by context and listener characteristics (Juslin et al., 2014). The social function of music is organised around self-identity, interpersonal relationships, and emotional regulation, which helps explain its centrality in everyday life (Juslin & Västfjäll, 2008; Van Goethem & Sloboda, 2011).

In this study, we focus specifically on theoretical and empirical work that conceptualises music listening as a form of mood or emotion regulation, rather than providing a general narrative of musical effects. Among the available frameworks, Saarikallio’s model and the seven factor Brief Music in Mood Regulation scale (B-MMR) (S. Saarikallio, 2012) are particularly suitable because they (a) were derived inductively from adolescents’ everyday music listening experiences (S. Saarikallio, 2007), (b) distinguish conceptually coherent strategies that map onto broader emotion regulation processes (e.g., distraction, expression, cognitive reflection) and (c) have been supported by a series of psychometric and validation studies in youth and young adult samples (S. Saarikallio, 2007; S. Saarikallio & Erkkilä, 2007; S. Saarikallio et al., 2014; S. H. Saarikallio et al., 2020). Other approaches capture broader motivations for music use or treat music simply as a stimulus that elicits emotions, but they do not offer the same level of specificity regarding how music is used to regulate mood in daily life. For these reasons, we consider the B-MMR a theoretically grounded and practically useful framework for examining music-based mood regulation among students.

Saarikallio’s model of music-based mood regulation identifies seven distinct strategies through which people use music in everyday life (S. Saarikallio, 2007; S. Saarikallio & Erkkilä, 2007): Entertainment (creation of a positive environment through music), Revival (recovering positive energy), Strong Sensation (increasing emotional intensity), Diversion (removing negative emotions), Discharge (emotional release), Mental Work (supporting concentration and reflection) and Solace (positive emotional refuge). The B-MMR operationalises these strategies as a 21-item self-report instrument, with three items per dimension. The original instrument demonstrated satisfactory internal consistency, with Cronbach’s alpha coefficients exceeding 0.73 across all dimensions, and provided adequate evidence of external validity through associations with established measures of emotion regulation. Because it combines a clear theoretical basis with a concise structure and captures everyday patterns of music use directly linked to emotion-regulation goals, the B-MMR has become a widely used measure in research on music, emotional functioning, and well-being.

### 1.2. The Psychophysiology of Music and Emotion

A growing body of research indicates that listening to music can support emotional regulation and well-being by shaping listeners’ psychophysiological responses to rhythmic, melodic, and harmonic elements (Brattico et al., 2011; Carlson et al., 2015; Hargreaves & Lamont, 2017; Schellenberg, 2012). Groarke and Hogan (2019) distinguish between musical self-selection—the ability to choose what to listen to—and the effects of that choice on emotional regulation, including nervousness, stress, tension, grief, and depression.

Different genres and musical styles are characterised by distinct patterns at the harmonic, melodic and rhythmic levels, which elicit physical, psychological and neural responses in listeners. Studies of emotional reactions to music show that the effects of listening are complex and depend on sound characteristics, technical elements of the music, associated memories and the context in which music is heard (Ansani et al., 2025; Baltazar et al., 2026; Eerola & Saari, 2025; Gomez-Canon et al., 2025; Juslin et al., 2015). Jimenez et al. (2020) investigate how music is used to evoke memories and recall and to elicit emotional reactions, highlighting the role of sound and harmonic components. The same musical style can have different emotional effects depending on listeners’ preferences and histories: negative emotions may arise in response to both beloved and disliked musical styles (Garrido & Schubert, 2015; Schubert, 2013). Cascarino et al. (2021) show that adolescents select musical styles and genres based on their affective impact on emotional well-being, and more recent work continues to examine these processes in diverse youth populations (Arthurs et al., 2025).

From this perspective, the emotional impact of music can be understood as listeners’ perceptions of sound frequencies and harmonic relations, which in turn give rise to affective responses. D. Willimek and Willimek’s (2011) theory of musical balance emphasises listeners’ desire for harmonic resolution, focusing on chords and progressions. According to this theory, harmonic structures possess an intrinsic emotional character across genres. From the standpoint of musical physics, the need for resolution is linked to the perception of continuity, suspension, and closure, which, in turn, depends on the patterning of consonances and dissonances. These structural properties support experiences of tension, release, and ecstasy in music, and empirical work has examined their effects across different styles (Graves et al., 2025; Eerola & Schutz, 2025; Krause et al., 2021a; Priyadarshini & Divakarla, 2026).

Neuroimaging studies have documented the involvement of brain regions and neuromodulatory systems associated with reward, motivation and emotion during music listening (Juslin et al., 2014; Juslin & Västfjäll, 2008; Koelsch, 2020; Lamont, 2011). Musical parameters such as tone, timbre, and pulse are crucial for eliciting emotions such as surprise, sadness, calm, and nostalgia (Chu & Tsai, 2026; Dai et al., 2025; Nawaz & Omigie, 2025). Brattico and Varankaitė (2019) describe the activation of neurotransmitters, such as dopamine, and hormones, such as oxytocin, linked to feelings of well-being during music listening, noting that activation patterns differ across styles and sound components. Increased activity in amygdala-related networks during music listening has been associated with the regulation of both positive and negative emotions (Ongchoco & Melcher, 2026). These findings support the view that music constitutes a multidimensional regulatory system in which cognitive appraisals, bodily arousal, affective meanings and contextual factors are dynamically integrated (Liljeström et al., 2013; Lundqvist et al., 2009; Sakka & Juslin, 2018).

At the same time, perception and cognition are central to understanding how music regulates affect. The emotional and psychological processes that occur during listening involve musical reception and perception (Tulving, 1983), in which affects (feelings, emotions and moods) are triggered as responses that go beyond purely intellectual processing. Music elicits emotional reactions that shape affect and behaviour, and empirical studies have examined how different musical manifestations affect emotion, behaviour and psychophysiology (McClymont & Krause, 2026; McKenzie et al., 2025; S. H. Saarikallio et al., 2019; Schellenberg, 2012; Schellenberg & Mankarious, 2012).

In this study, we draw on this integrative perspective to position the B-MMR as an instrument that captures patterns of music-based regulatory strategy use grounded in these theoretical frameworks. However, we do not test neurobiological or psychophysiological mechanisms directly, as this would require experimental manipulation and multi-method assessment beyond the scope of this initial psychometric validation.

### 1.3. The Current Study

Building on the integrative evidence reviewed, the current study advances a theoretically grounded hypothesis that moves beyond a merely descriptive link between music and emotional regulation. Specifically, the convergence of (i) music’s functional role in self-regulation strategies (S. Saarikallio, 2007; S. H. Saarikallio et al., 2020), (ii) the differential affective affordances of musical structures and genres (Juslin et al., 2015; B. Willimek & Willimek, 2022), and (iii) their neuropsychological correlates within emotion-related brain systems (Brattico & Varankaitė, 2019; Koelsch, 2020) suggests that music functions as a multidimensional regulatory system in which cognitive, affective, and contextual processes are dynamically integrated.

Despite growing interest in music-based mood regulation, there remains a lack of validated Portuguese instruments for assessing how individuals use music to regulate their mood. This gap limits the inclusion of music-based regulation in behavioural, educational and health research with Portuguese-speaking populations and hinders cross-cultural comparisons with studies conducted in other languages. Adapting and validating a Portuguese version of the B-MMR is therefore an important, albeit incremental, step for the field, providing a psychometrically robust tool to support future work on music, emotion regulation, and well-being in Portuguese contexts.

Building on the theoretical and empirical background outlined above, the following hypotheses were formulated:

**H1**  (Factorial validity)**.** *The Portuguese version will replicate the original seven-factor structure.*

**H2**  (Reliability)**.** *All dimensions will show adequate internal consistency and composite reliability.*

**H3**  (Measurement invariance)**.** *The factorial structure will be invariant across sex.*

**H4**  (Preliminary construct-related evidence)**.** *B-MMR dimensions will show theoretically predictable associations with corresponding emotional experiences during music listening.*

## 2. Materials and Methods

### 2.1. Participants

The sample comprised 493 students from Portugal, recruited through heterogeneous purposive sampling (Memon et al., 2025). Participants were recruited through a combination of institutional collaborations and online dissemination. Specifically, secondary schools and universities across Portugal were contacted and invited to participate. In addition, the survey was distributed through academic mailing lists and student networks. This multi-channel recruitment strategy aimed to increase heterogeneity and reduce selection bias within the constraints of non-probabilistic sampling. Due to the administrative and bureaucratic complexities of collecting data in schools in Portugal, including schools’ availability amid multiple similar requests and the requirement for parental or legal guardian consent for children under 18, the authors decided to extend the period to obtain a larger sample. This sampling strategy was deemed suitable given pronounced interindividual differences in musical perception and emotional responsiveness, the exploratory nature of the validation, and limited access to the population of interest.

Regarding sex identification, 189 participants reported being men (38.3%), 291 women (59.0%), and 8 indicated “other/prefer not to say” (1.6%). Participants’ ages were categorised as follows: 16–18 years (56.0%), 19–25 years (38.9%), and 26 years or older (5.1%). All respondents were enrolled in Portuguese educational institutions, with 51.3% attending secondary education (10th–12th grades) and 48.7% pursuing higher education. The presence of participants aged 26 and older (5.1% of the sample) reflects the inclusion of mature students and non-traditional learners in higher education, consistent with Portugal’s educational landscape, where adults pursue degrees alongside traditional-aged students. All 493 participants were included in all analyses.

### 2.2. Instruments

A Portuguese version of the Brief Music in Mood Regulation (B-MMR) scale (S. Saarikallio, 2012) was employed in the present study. The B-MMR is a domain-specific measure of music-based emotion regulation that captures how individuals use music to pursue regulatory goals (e.g., enhancing positive affect, distracting from negative thoughts, finding solace) in everyday contexts. This self-administered measure comprises 21 items organised into seven dimensions (Entertainment, Revival, Strong Sensation, Diversion, Discharge, Mental Work, Solace), each represented by three items. Responses to the B-MMR items were recorded on a five-point Likert scale, ranging from 1 (strongly disagree) to 5 (strongly agree).

In addition, participants were asked to respond to the question “How do you feel when listening to music?” by rating the intensity of seven basic emotions, as conceptualised by Ekman and Cordaro (2011): anger, fear, surprise, sadness, disgust, contempt, and happiness, on a scale from 1 (not at all) to 5 (very much). Basic emotion ratings were used as external criteria for three reasons: (1) minimising respondent burden (comprehensive emotion regulation batteries would substantially increase survey length and attrition risk); (2) adopting an incremental validation approach focused on establishing core psychometric properties before comprehensive construct validation; and (3) examining affective experiences as proximal, ecologically relevant outcomes of music-based regulatory strategies. We acknowledge that this provides limited, indirect evidence of construct validity, as emotion ratings index affective states rather than regulatory processes. Future validation with established emotion regulation measures (DERS-26, ERQ, CERQ) is a priority.

### 2.3. Procedure

The adaptation of the instrument into Portuguese followed established methodological standards for test translation and cultural adaptation, drawing on the procedures outlined by Balluerka et al. (2007) and Muñiz et al. (2013), as well as the recommendations of the International Test Commission guidelines (Hernández et al., 2020). Initially, the original English items were translated into Portuguese and then back-translated into English. These translations were carried out independently by two bilingual researchers with demonstrated proficiency in both languages. No significant discrepancies were found between the translators regarding the wording or meaning of the items. An expert panel composed of specialists in musicology and psychology then evaluated the translated items to ensure semantic clarity, conceptual equivalence, and fidelity to the original content. Following expert review, cognitive interviews were conducted with a pilot group of 11 university students (6 women, 5 men; age 20–24) to assess item comprehension, identify potential ambiguities, and evaluate the cultural appropriateness of the translated items. Participants were asked to complete the questionnaire while thinking aloud, verbalising their interpretation of each item. The interviewer then probed specific items to assess whether students understood them as intended and whether any phrasing seemed unclear or culturally inappropriate. They were not included in the final sample. Based on this feedback, no substantive changes were required, confirming adequate comprehension across items. Following this review, the instrument was administered for empirical validation.

Prior to data collection, the study received ethical approval from the Ethics Committee of the School of Social Sciences and Humanities at NOVA University of Lisbon (CE-NOVA_FCSH_2024/63, 15 July 2024) and from the Portuguese Ministry of Education (Inquiry No. 1471100001, 3 September 2024). Subsequently, the required sample size to ensure adequate representativeness of the Portuguese student population was calculated using Cochran’s (2007) formula for infinite populations, yielding a minimum target of 400 participants. Data were collected via an online questionnaire. Eligibility criteria included (a) current enrolment in a formal educational institution in Portugal and (b) a minimum age of 16 years.

Data collection took place between November 2024 and November 2025. This extended 12-month recruitment window reflects the administrative and bureaucratic complexity of obtaining institutional approvals and coordinating data collection in Portuguese educational institutions. This process requires school board availability, alignment with academic calendars to avoid examination periods, and parental consent for participants under 18 years. This extended period enabled achieving the target sample size (*n* = 493) and ensured adequate heterogeneity in age and educational level, though it introduces potential temporal variability, which is acknowledged as a limitation.

Before participating, respondents were informed of the study’s anonymous, voluntary nature, their right to withdraw at any time without penalty, and the confidential handling of their data in accordance with Regulation (EU) 2016/679 and its transposition into Portuguese law through Law No. 58/2019 of 8 August. Written informed consent was obtained from all participants, and from their legal guardians for those under 18. The study was conducted in full compliance with the ethical principles of the Declaration of Helsinki.

### 2.4. Data Analysis

All statistical analyses were conducted using IBM SPSS Statistics (Version 25), IBM AMOS (Version 22) and R lavaan package (Version 0.6-21, December 2025. Initially, descriptive statistics were computed for each of the 21 questionnaire items. Subsequently, a confirmatory factor analysis (CFA) was conducted. Model parameters were estimated using maximum-likelihood estimation, which is appropriate under conditions of approximate multivariate normality (Brown, 2006). Model fit was evaluated using multiple complementary indices, including the Comparative Fit Index (CFI), the Tucker–Lewis Index (TLI), and the Root Mean Square Error of Approximation (RMSEA). Following commonly recommended values, acceptable model fit was indicated by CFI and TLI ≥ 0.90 and RMSEA ≤ 0.08. The reliability of the latent factors derived from the measurement model was then examined using two complementary indicators: internal consistency, assessed through Cronbach’s alpha coefficients, and composite reliability indices. To be considered adequate, both estimators had to equal or exceed the 0.7 threshold. In addition, the average variance extracted (AVE) for each factor was calculated to evaluate both convergent and discriminant validity. For convergent validity, the AVE of a construct had to be greater than 0.5, while for discriminant validity, the AVE of each construct had to be greater than the square of the correlations between the construct and other latent variables of the model.

Measurement invariance of the factor structure was subsequently tested using a multigroup analysis, with sex specified as a moderator. Two groups were compared: men (38.3% of the sample) and women (59.0%). A complete four-step measurement invariance sequence was carried out: configural, metric, scalar, and full uniqueness.

Finally, correlations with basic emotions during music listening were analysed as preliminary construct-based evidence, consistent with prior work linking B-MMR scores to affective experiences and emotion-regulation outcomes.

Spearman’s rank-order correlation was used because several variables violated the assumption of univariate normality, even though the overall data set demonstrated multivariate normality.

## 3. Results

### 3.1. Descriptive Statistics

Table 1 summarises the descriptive statistics for the Portuguese version of the B-MMR scale items. Item means (*M*) ranged from 2.36 for Item 14 to 4.69 for Item 2, and the largest standard deviation (*SD*) was 1.44 for Item 13. The distributional properties indicated negative skewness for most items (*n* = 19) and negative kurtosis for most items (*n* = 15), revealing no problematic results. In addition, Table 1 reports the standardised factor loadings (*λ*) for the 21 items, as estimated in the confirmatory factor analysis model.

### 3.2. Confirmatory Analysis

**Figure 1:**
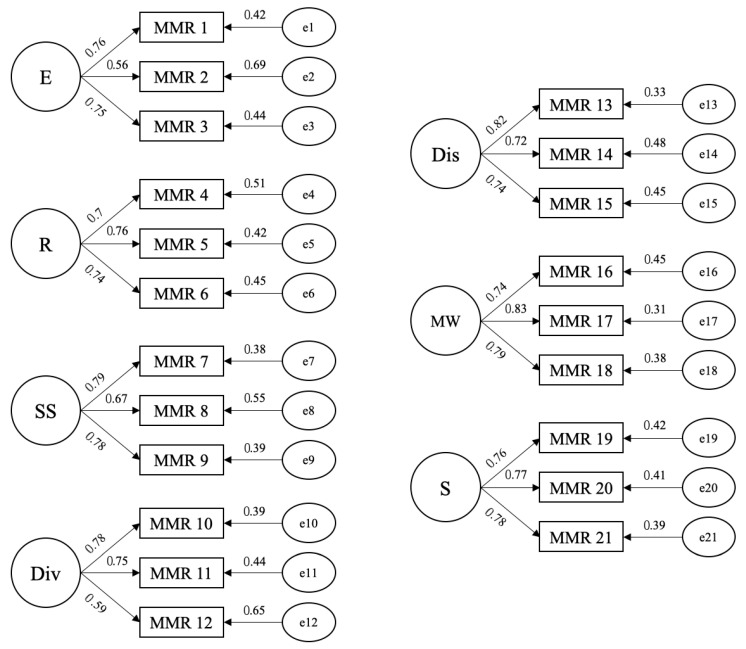
Factor model of the Portuguese adaptation of the B-MMR. NOTE. E = Entertainment; R = Revival; SS = Strong Sensation; Div = Diversion; Dis = Discharge; MW = Mental Work; S = Solace.

The seven-factor structure (S. Saarikallio, 2012), comprising three indicators per factor, was successfully replicated. All factor loadings and error variances were statistically significant (*p* < 0.001) and adequate in magnitude (Figure 1). Model fit statistics (Table 2) indicated acceptable fit. Modification indices were inspected but did not reveal any theoretically justified adjustments. Alternative models were not tested, as the study’s objective was to replicate the original seven-factor structure to maintain cross-cultural comparability with validated B-MMR versions (Muñiz et al., 2013).

### 3.3. Reliability and Average Variance Extracted

As presented in Table 3, all reliability indices exceeded the recommended threshold of 0.7. The table also reports the average variance extracted (AVE) for each factor, with values above 0.5 for all but one factor; this exception yielded an AVE of 0.48, which would correspond to 0.5 when rounded to one decimal place.

For each B-MMR dimension, except Entertainment, the average variance extracted (AVE) exceeded the squared correlations with the other latent constructs. As shown in Table 4, all Spearman rank-order correlations between factors were lower than the square root of the corresponding AVE values, supporting adequate discriminant validity.

### 3.4. Invariance

Table 5 presents the model fit indices for the four-step measurement invariance sequence across sex. Step 1 (configural invariance) evaluated whether the same factor structure held in both groups, with all parameters freely estimated. Step 2 (metric invariance) further constrained factor loadings to be equal across groups. Step 3 (scalar invariance) further constrained item intercepts to be equal, and Step 4 (full uniqueness invariance) further constrained residual variances across groups. Model comparisons relied on the criteria proposed by Chen (2007): a change in CFI (ΔCFI) no greater than −0.010 and a change in RMSEA (ΔRMSEA) no greater than 0.015 indicate acceptable invariance at each step.

As shown in Table 5, the configural model (M1) demonstrated acceptable fit, confirming that the seven-factor structure of the B-MMR is replicable across sex groups. Metric invariance (M2) was supported: constraining factor loadings to equality across groups produced no meaningful deterioration in fit, indicating that items relate to their respective latent factors with equivalent strength for men and women. Scalar invariance (M3) was likewise supported: the additional equality constraints on item intercepts did not produce a meaningful change in fit according to Chen’s (2007) criteria. Scalar invariance implies that item intercepts, that is, the mean levels at which items are endorsed, are equivalent across sex, supporting the meaningful comparison of latent factor means between men and women. Full uniqueness (strict) invariance (M4) also met the ΔCFI criterion, indicating that residual variances are comparable across groups, though this most restrictive level of invariance is not required for group comparisons of latent means or scale scores.

### 3.5. External Validity: Associations with Emotional Experiences

To provide preliminary, exploratory evidence of construct-related validity, Spearman correlation coefficients were computed between the dimensions of the Portuguese version of the B-MMR and the basic emotions (anger, fear, surprise, sadness, disgust, contempt, and happiness) (Table 6). The findings indicate several statistically significant associations, generally weak to moderate in strength. Sadness was positively and significantly associated with all questionnaire factors. Likewise, contempt and happiness showed significant correlations with all but one factor, whereas the remaining emotions demonstrated significant relationships with at least two of the instrument’s dimensions.

## 4. Discussion

The present study aimed to translate, adapt, and psychometrically evaluate the Brief Music in Mood Regulation (B-MMR) scale (S. Saarikallio, 2012) for use among a Portuguese student population. Overall, the findings demonstrated satisfactory psychometric properties in a student sample, though with important qualifications regarding construct validation and generalisability. All four formulated hypotheses were supported and are discussed below, along with the study’s limitations and future research directions.

### 4.1. Summary of Main Findings

Regarding Hypothesis 1 (Factorial validity), replicating the original factor structure yielded satisfactory fit indices according to the criteria proposed by Schermelleh-Engel et al. (2003) at the global level and for individual parameters (Hu & Bentler, 2009). The indices observed in the Portuguese version are comparable to those reported for the original English-language scale. This result aligns with theoretical perspectives that conceptualise music listening as a multidimensional regulatory activity serving several psychosocial functions, including identity construction, interpersonal connection, and emotional self-management. In line with research highlighting adolescents’ and young adults’ intensive and functionally diverse engagement with music (e.g., Elvers et al., 2018; Krause et al., 2015, 2021b, 2023; S. H. Saarikallio et al., 2020), the current findings indicate that these functions can be meaningfully captured in a new linguistic and cultural context.

Regarding Hypothesis 2 (Reliability), reliability estimates were adequate across all factors, as evidenced by internal consistency indicators (Bonett & Wright, 2015) and composite reliability (Carmines & Zeller, 1979; Nunnally, 1978). The estimates are comparable to those reported in the original B-MMR validation (S. Saarikallio, 2012: α range 0.73–0.82), suggesting that translation into Portuguese did not compromise measurement precision. Furthermore, the average variance extracted (AVE) values provided compelling evidence of convergent and discriminant validity (Fornell & Larcker, 1981). For convergent validity, all but one factor accounted for more than 50% of the variance in their respective indicators, with the remaining variance attributable to measurement error. The Entertainment factor yielded an AVE of 0.48, falling slightly below the conventional 0.50 threshold. This represents a minor psychometric weakness that warrants acknowledgement. Yet, overall evidence supports retaining this dimension since it demonstrates adequate reliability (α = 0.72, CR = 0.73) and acceptable factor loadings (0.56–0.76), its conceptual content is theoretically coherent and central to the B-MMR framework, it shows appropriate discriminant validity and theoretically consistent correlations with external criteria, and removing it would compromise the theoretical completeness of the seven-factor model validated across multiple studies (S. Saarikallio, 2012; Ansani et al., 2025). Regarding discriminant validity, each construct shared more variance with its own indicators than with any other latent factor in the model. This suggests that, although the strategies are interrelated, they represent distinguishable regulatory functions rather than a single, undifferentiated emotional regulation tendency. Such differentiation is important for behavioural research because it enables the examination of specific patterns of music use, such as whether reliance on Discharge or Solace is associated with adaptive versus maladaptive outcomes, rather than treating music listening as a unitary construct.

Regarding Hypothesis 3 (Measurement invariance), the complete four-step measurement invariance sequence supported configural, metric, and scalar invariance of the B-MMR across gender groups, in line with the criteria proposed by Chen (2007) and Cheung and Rensvold (2002). The factor structure was replicated across groups (configural invariance), factor loadings were equivalent across groups (metric invariance), and item intercepts were also equivalent (scalar invariance). Changes in CFI and RMSEA remained within acceptable limits at each step. Scalar invariance is the minimum level required to justify meaningful comparisons of latent factor means or observed scale scores between men and women (Vandenberg & Lance, 2000), and the present data meet this standard. Full uniqueness invariance (Step 4) was also supported by CFI-based criteria. These findings confirm that the Portuguese B-MMR functions equivalently across sex, providing researchers with confidence that observed group differences in B-MMR subscale scores reflect true differences in music-based mood regulation strategies rather than measurement artefacts.

Regarding Hypothesis 4 (Preliminary construct-related evidence), all B-MMR dimensions were positively associated with at least two of Ekman and Cordaro’s (2011) basic emotions when participants reported how they felt while listening to music. Significant positive correlations between sadness and all seven dimensions suggest that individuals who use music intensively for mood regulation tend to experience sadness more strongly during music listening, whether because they turn to music in low moods, choose pieces that match their affect, or engage in reflective listening that makes sadness more salient. Positive links between Discharge and Strong Sensation and higher levels of negative emotions (e.g., anger, contempt) are consistent with the idea that these strategies involve seeking intense or cathartic musical experiences to express and process difficult feelings. Conversely, systematic associations between several B-MMR dimensions and happiness are compatible with the view that many regulatory uses of music (e.g., Entertainment, Revival and Solace) aim to enhance or restore positive affect. These findings align with evidence that music can mobilise a wide range of affective states, including both positive and negative emotions, and that listeners frequently use music strategically to modulate these states (e.g., Garrido & Schubert, 2015; Juslin et al., 2014; Koelsch, 2020). They are also broadly consistent with the theoretical content of the B-MMR strategies. Given small to moderate effect sizes and the indirect nature of emotion ratings as evidence of regulatory processes, these results should be interpreted cautiously.

### 4.2. Limitations

Several limitations should be acknowledged. The sample comprised students recruited through purposive sampling, which limits the generalisability of the findings to other population segments. Potential sources of bias include self-selection bias, given that participation was voluntary, and the exclusive reliance on self-report measures, which may be influenced by social desirability and recall biases.

In addition, several sample and procedural characteristics constrain the scope of the present findings. The age range was limited to older adolescents and adults enrolled in secondary and higher education, so the factor structure and functioning of the Portuguese B-MMR cannot be assumed to generalise to younger adolescents, older adults, or individuals outside formal education. The sample was also educationally specific, consisting entirely of students, which may be associated with patterns of music use and mood regulation linked to academic demands and student lifestyles.

Furthermore, all measures were administered online, which, although convenient and consistent with current research practices, may introduce variability in response quality due to differences in devices, environments, and levels of attention. While this extended period introduces potential temporal variability (e.g., seasonal effects, cohort differences), we have no reason to believe that music-listening habits or the B-MMR’s psychometric properties would change substantially over this timeframe. Future research should test measurement invariance across data collection periods to rule out temporal artefacts. The consistent online administration format and identical item wording throughout data collection minimised procedural variability. These features should be considered when interpreting the results and highlight the need for future studies with more diverse recruitment strategies and alternative administration formats.

Another constraint of the present study concerns the scope of the external validity assessment. Specifically, self-reported emotional experiences were used as external criteria, which, while informative, do not directly index emotion regulation processes. As a result, the findings offer only indirect evidence of convergence with established emotion regulation constructs. 

### 4.3. Future Research Directions

Future research should evaluate the structure and functioning of the Portuguese B-MMR in community and clinical samples, including older adults, to determine whether the factor configuration and psychometric properties generalise across age groups and contexts. To achieve this, measurement invariance analyses across additional sociodemographic variables (e.g., age groups, educational levels, clinical versus non-clinical status) would be valuable. Moreover, all variables were assessed via self-report, which may be influenced by recall biases and social desirability.

Future studies could build on these results by incorporating well-validated instruments such as the Brief Version of the Difficulties in Emotion Regulation Scale (DERS-26; Bjureberg et al., 2016). In addition, incorporating behavioural indicators, physiological measures, or neurobiological indices would provide a more comprehensive assessment of the B-MMR’s convergent validity, in line with multi-method approaches in the behavioural sciences.

Results point to potential applications for behavioural research and practice. First, the Portuguese B-MMR offers a brief instrument that can be integrated into broader studies of stress, coping, and well-being among adolescents and young adults, where music is already a prevalent everyday activity. Second, the differentiation among strategies such as Entertainment, Revival, Solace, and Discharge may help identify profiles of music use associated with more adaptive or more problematic emotional regulation. For example, a profile characterised by frequent use of Solace and Mental Work in the context of adversity may indicate reflective, meaning-focused coping, whereas a predominant reliance on Discharge under high negative affect might be linked to less adaptive outcomes, as suggested by prior work on emotion regulation and music. Such profiles could inform psychoeducational or preventive interventions that promote more flexible and constructive uses of music in everyday emotional life and well-being.

Given that the present study is cross-sectional and relies exclusively on self-report data, such profiles cannot be inferred from our findings and remain a topic for future research rather than established conclusions. Longitudinal and intensive repeated-measures designs (e.g., experience sampling) could clarify whether specific B-MMR strategies predict trajectories of well-being, stress, or psychopathology over time, and whether changes in music-based regulation co-occur with changes in broader emotion-regulation repertoires. Test-retest reliability assessment over appropriate intervals (e.g., 2–4 weeks) would establish whether the Portuguese B-MMR captures stable individual differences in mood regulation strategies or is sensitive to short-term state variations.

## 5. Conclusions

This study presents a Portuguese adaptation of the Brief Music in Mood Regulation (B-MMR) scale and provides initial psychometric evidence supporting its use in student samples. It extends previous research by replicating the seven-factor structure, reliability, and measurement invariance of the B-MMR in a new cultural-linguistic setting. At the same time, the findings should be treated as preliminary rather than definitive. The external validity results are based on indirect indicators of emotional experience, and the sample is limited to non-clinical students. Consequently, the Portuguese B-MMR should be considered a promising instrument for research in similar populations, rather than a fully generalisable measure across all Portuguese-speaking groups. Further validation is needed in larger, more diverse samples, including clinical and at-risk populations, and in studies that incorporate established emotion-regulation measures and employ multi-method designs. Such work will be essential to confirm and extend the present results before the scale is used for broader diagnostic or intervention purposes.

## Figures and Tables

**Table 1:** Descriptive statistics and factor load of B-MMR items.

Items	*M*	*SD*	Asymmetry	Kurtosis	*λ*
MMR 1 When I’m busy around the house and no one else is around, I like to have some music on the background [Quando estou ocupado/a por casa e não há ninguém por perto, gosto de ouvir música de fundo.]	4.5	0.85	−2.08	4.66	0.76
MMR 2 I listen to music to make cleaning and doing other housework more pleasant [Oiço música para tornar as limpezas e outras tarefas domésticas mais agradáveis.]	4.69	0.79	−2.7	7.89	0.56
MMR 3 I usually put background music on to make the atmosphere more pleasant [Costumo pôr música de fundo para tornar o ambiente mais agradável.]	4.19	0.99	−1.19	0.93	0.75
MMR 4 When I’m tired out, I rest by listening to music [Quando me sinto cansado/a, oiço música para descansar.]	3.41	1.3	−0.34	−0.07	0.7
MMR 5 I listen to music to perk up after a rough day [Oiço música para me animar depois de um dia difícil.]	3.97	1.15	−0.95	−0.01	0.76
MMR 6 When I’m exhausted, I listen to music to perk up [Quando estou exausto/a, ouço música para me arrebitar.]	3.37	1.29	−0.25	−1.07	0.74
MMR 7 I feel fantastic putting my soul fully into the music [Sinto-me fantástico/a quando me entrego inteiramente à música.]	4.21	1.01	−1.17	0.69	0.79
MMR 8 Music has offered me magnificent experiences [A música já me proporcionou experiências magníficas.]	4.56	0.79	−1.96	3.74	0.67
MMR 9 I want to feel the music in my whole body [Quero sentir a música no meu corpo todo.]	3.79	1.24	−0.71	−0.57	0.78
MMR 10 When stressful thoughts keep going round and round in my head, I start to listen to music to get them off my mind [Quando não paro de remoer pensamentos stressantes, oiço música para os tirar da cabeça.]	3.62	1.27	−0.58	−0.72	0.78
MMR 11 For me, music is a way to forget about my worries [Para mim, a música é uma forma de esquecer as minhas preocupações.]	3.77	1.19	−0.72	−0.44	0.75
MMR 12 When I feel bad, I try to get myself in a better mood by engaging in some nice, music-related activity [Quando me sinto em baixo, tento ficar mais bem-disposto/a fazendo alguma atividade agradável relacionada com a música.]	3.61	1.17	−0.47	−0.66	0.59
MMR 13 When I’m angry with someone, I listen to music that expresses my anger [Quando estou zangado/a com alguém, oiço música que exprime a minha raiva.]	2.81	1.44	0.2	−1.3	0.82
MMR 14 When I’m really angry, I feel like listening to some aggressive music [Quando estou muito zangado/a, ponho-me a ouvir música mais agressiva.]	2.36	1.4	0.61	−0.97	0.72
MMR 15 When everything feels bad, it helps me to listen to music that expresses my bad feelings [Quando tudo me corre mal, ajuda-me ouvir música que exprime os meus sentimentos negativos.]	3.29	1.42	−0.3	−1.19	0.74
MMR 16 Music has helped me to get through hard experiences [A música ajudou-me a ultrapassar experiências difíceis.]	3.84	1.23	−0.8	−0.4	0.74
MMR 17 Music helps me to recognize different feelings in myself [A música ajuda-me a compreender diferentes sentimentos em mim próprio/a.]	3.63	1.29	−0.62	−0.7	0.83
MMR 18 When I’m distressed by something, music helps me to clarify my feelings [Quando estou angustiado/a com alguma coisa, a música ajuda-me a perceber melhor o que sinto.]	3.22	1.3	−0.19	−1.04	0.79
MMR 19 I listen to music to find solace when worries overwhelm me [Oiço música para me acalmar sempre que as preocupações me atormentam.]	3.49	1.22	−0.37	−0.85	0.76
MMR 20 When everything feels bad, music understands and comforts me [Quando tudo corre mal, a música compreende-me e reconforta-me.]	3.61	1.27	−0.6	−0.67	0.77
MMR 21 When I’m feeling sad, listening to music comforts me [Quando me sinto triste, ouvir música reconforta-me.]	4.08	1.06	−1.06	0.41	0.78

Note. Standard error for asymmetry = 0.11; Standard error for kurtosis = 0.22; *λ* = factor loading.

**Table 2:** B-MMR goodness-of-fit indices.

Model	X^2^	*df*	X^2^/*df*	NFI	TLI	CFI	RMSEA (IC 90%)
21 items	449.66 *	168	2.68	0.91	0.92	0.94	0.06 (0.05, 0.07)

Note. *df* = Degrees of freedom; NFI = Normed Fit Index; TLI = Tucker-Lewis Index; CFI = Comparative Fit Index; RMSEA = Root Mean Square Error of Approximation. * *p* < 0.001.

**Table 3:** Reliability Indices and Average Extracted Variance (AVE) for the B-MMR.

Estimation Method	E	R	SS	Div	Dis	MW	S
α by Cronbach	0.72	0.78	0.77	0.73	0.8	0.82	0.81
Composite reliability	0.73	0.78	0.79	0.75	0.8	0.83	0.81
AVE	0.48	0.54	0.56	0.51	0.58	0.62	0.59

NOTE. E = Entertainment; R = Revival; SS = Strong Sensation; Div = Diversion; Dis = Discharge; MW = Mental Work; S = Solace.

**Table 4:** Correlations among B-MMR factors versus the square root of the AVE.

	E	R	SS	Div	Dis	MW	S
E	0.69						
R	0.46	0.73					
SS	0.36	0.27	0.75				
Div	0.45	0.57	0.4	0.71			
Dis	0.31	0.35	0.35	0.34	0.76		
MW	0.39	0.48	0.57	0.56	0.56	0.79	
S	0.46	0.56	0.46	0.69	0.53	0.72	0.77

NOTE. E = Entertainment; R = Revival; SS = Strong Sensation; Div = Diversion; Dis = Discharge; MW = Mental Work; S = Solace; All correlations were significant at the 0.01 level (bilateral).

**Table 5:** Four-step measurement invariance analysis by sex.

Model	χ^2^	*df*	χ^2^/*df*	CFI	TLI	RMSEA (90% CI)	ΔCFI	ΔRMSEA
M1. Configural	684.20 *	336	2.04	0.930	0.912	0.066 [0.059, 0.073]	—	—
M2. Metric	696.27 *	350	1.99	0.930	0.916	0.064 [0.057, 0.071]	0.0004	−0.002
M3. Scalar	720.09 *	364	1.98	0.928	0.917	0.064 [0.057, 0.071]	−0.002	−0.001
M4. Full Uniqueness	769.15 *	385	2.00	0.923	0.915	0.064 [0.058, 0.071]	−0.006	0.001

Note. *df* = Degrees of freedom; CFI = Comparative Fit Index; TLI = Tucker-Lewis Index; RMSEA = Root Mean Square Error of Approximation. CI = confidence interval; ΔCFI and ΔRMSEA = change in fit index relative to the preceding model; * *p* < 0.001—indicates not applicable (no comparison model exists for the configural model).

**Table 6:** External Validity Analysis with Basic Emotions.

	Anger	Fear	Surprise	Sadness	Disgust	Contempt	Happiness
E	−0.05	−0.07	−0.02	0.2 **	−0.12 *	0.07	0.25 **
R	0.09	0.01	0.12 **	0.14 **	−0.02	0.23 **	0.23 **
SS	0.1 *	0.11 *	0.26 **	0.21 **	0.04	0.24 **	0.24 **
Div	0.01	−0.04	0.09	0.14 **	−0.11 *	0.17 **	0.29 **
Dis	0.35 **	0.12 **	0.09 *	0.38 **	0.1 *	0.22 **	0.08
MW	0.21 **	0.06	0.23 **	0.29 **	0.04	0.27 **	0.18 **
S	0.1*	0.01	0.1 *	0.26 **	−0.07	0.19 **	0.28 **

* Correlation is significant at the −0.05 level (bilateral). ** Correlation is significant at the 0.01 level (bilateral) NOTE. E = Entertainment; R = Revival; SS = Strong Sensation; Div = Diversion; Dis = Discharge; MW = Mental Work; S = Solace.

## Data Availability

The data that support the findings of this study are available from the corresponding author upon reasonable request.

## References

[B1-behavsci-16-00761] Ansani A., Mallia L., Saarikallio S. (2025). Music in mood regulation brief scale (B-MMR): Italian version and insights on musical preferences, expertise, and reward experiences. Music Perception.

[B2-behavsci-16-00761] Arthurs Y., Merlini E., Omigie D. (2025). Unpacking musical beauty: Sound, emotion, and impact differences across expertise and personality. PLoS ONE.

[B3-behavsci-16-00761] Balluerka N., Gorostiaga A., Alonso-Arbiol I., Haranburu M. (2007). The adaptation of measurement instruments from one culture to another: A practical perspective. Psicothema.

[B4-behavsci-16-00761] Baltazar M., Burunat I., Saarikallio S. (2026). The emotional complexity of musical experiences: Cultural and individual factors. Journal of Research in Personality.

[B5-behavsci-16-00761] Baltazar M., Västfjäll D., Asutay E., Koppel L., Saarikallio S. (2019). Is it me or the music? Stress reduction and the role of regulation strategies and music. Music & Science.

[B6-behavsci-16-00761] Bjureberg J., Ljótsson B., Tull M. T., Hedman E., Sahlin H., Lundh L. G., Bjärehed J., DiLillo D., Messman T. L., Hellner C., Gratz K. L. (2016). Development and validation of a brief version of the difficulties in emotion regulation scale: The DERS-16. Journal of Psychopathology and Behavioral Assessment.

[B7-behavsci-16-00761] Bonett D. G., Wright T. A. (2015). Cronbach’s alpha reliability: Interval estimation, hypothesis testing, and sample size planning. Journal of Organizational Behavior.

[B8-behavsci-16-00761] Brattico E., Alluri V., Bogert B., Jacobsen T., Vartiainen N., Nieminen S., Tervaniemi M. (2011). A Functional MRI study of happy and sad emotions in music with and without lyrics. Frontiers in Psychology.

[B9-behavsci-16-00761] Brattico E., Varankaitė U. (2019). Aesthetic empowerment through music. Musicae Scientiae.

[B10-behavsci-16-00761] Brown T. A. (2006). Confirmatory factor analysis for applied research.

[B11-behavsci-16-00761] Carlson E., Saarikallio S., Toiviainen P., Bogert B., Kliuchko M., Brattico E. (2015). Maladaptive and adaptive emotion regulation through music: A behavioral and neuroimaging study of males and females. Frontiers in Human Neuroscience.

[B12-behavsci-16-00761] Carmines E. G., Zeller R. A. (1979). Reliability and validity assessment.

[B13-behavsci-16-00761] Cascarino E., Knight T., Macdonald J. A. (2021). “The music gets through”: Experiences of classical composers and observations of hospitalized adolescents in a music-based intervention. Psychology of Music.

[B14-behavsci-16-00761] Chen F. F. (2007). Sensitivity of goodness of fit indexes to lack of measurement invariance. Structural Equation Modeling: A Multidisciplinary Journal.

[B15-behavsci-16-00761] Cheung G. W., Rensvold R. B. (2002). Evaluating goodness-of-fit indexes for testing measurement invariance. Structural Equation Modeling: A Multidisciplinary Journal.

[B16-behavsci-16-00761] Chu T., Tsai C. (2026). Music’s context-dependent influence on oxytocin, social bonding, and emotion regulation: A systematic review. Frontiers in Cognition.

[B17-behavsci-16-00761] Cochran W. G. (2007). Sampling techniques.

[B18-behavsci-16-00761] Dai R., Toiviainen P., Campo F. F., Brattico E. (2025). Differences in dynamic functional connectivity between musicians and non-musicians during naturalistic music listening. Frontiers in Neuroscience.

[B19-behavsci-16-00761] Eerola T., Saari P. (2025). What emotions does music express? structure of affect terms in music using iterative crowdsourcing paradigm. PLoS ONE.

[B20-behavsci-16-00761] Eerola T., Schutz M. (2025). Major-minorness in tonal music: Evaluation of relative mode estimation using expert ratings and audio-based key-finding principles. Psychology of Music.

[B21-behavsci-16-00761] Ekman P., Cordaro D. (2011). What is meant by calling emotions basic. Emotion Review.

[B22-behavsci-16-00761] Elvers P., Fischinger T., Steffens J. (2018). Music listening as self-enhancement: Effects of empowering music on momentary explicit and implicit self-esteem. Psychology of Music.

[B23-behavsci-16-00761] Fornell C., Larcker D. F. (1981). Evaluating structural equation models with unobservable variables and measurement error. Journal of Marketing Research.

[B24-behavsci-16-00761] Garrido S., Schubert E. (2015). Moody melodies: Do they cheer us up? A study of the effect of sad music on mood. Psychology of Music.

[B25-behavsci-16-00761] Gomez-Canon J. S., Lennie T. M., Eerola T., Aragon P., Cano E., Herrera P., Gomez E. (2025). Personalisation and profiling using algorithms and not-so-popular colombian music: Goal-directed mechanisms in music emotion recognition. EPJ Data Science.

[B26-behavsci-16-00761] Graves T. A., Eerola T., Clayton M., Nizami S. M., Rafiq M. U. (2025). A genre-specific structure of subjective feeling in music listening: What do *qawwālī* listeners feel?. Musicae Scientiae.

[B27-behavsci-16-00761] Groarke J. M., Hogan M. J. (2019). Listening to self-chosen music regulates induced negative affect for both younger and older adults. PLoS ONE.

[B28-behavsci-16-00761] Hargreaves D., Lamont A. (2017). The psychology of musical development..

[B29-behavsci-16-00761] Harley J. M., Pekrun R., Taxer J. L., Gross J. J. (2019). Emotion regulation in achievement situations: An integrated model. Educational Psychologist.

[B30-behavsci-16-00761] Hernández A., Hidalgo M. D., Hambleton R. K., Gómez-Benito J. (2020). International test commission guidelines for test adaptation: A criterion checklist. Psicothema.

[B31-behavsci-16-00761] Hofmann W., Schmeichel B. J., Baddeley A. D. (2012). Executive functions and self-regulation. Trends in Cognitive Sciences.

[B32-behavsci-16-00761] Hu L. T., Bentler P. M. (2009). Cutoff criteria for fit indexes in covariance structure analysis: Conventional criteria versus new alternatives. Structural Equation Modeling: A Multidisciplinary Journal.

[B33-behavsci-16-00761] Jimenez I., Kuusi T., Doll C. (2020). Common chord progressions and feelings of remembering. Music & Science.

[B34-behavsci-16-00761] Juslin P. N. (2013). From everyday emotions to aesthetic emotions: Towards a unified theory of musical emotions.

[B35-behavsci-16-00761] Juslin P. N., Barradas G., Eerola T. (2015). From sound to significance: Exploring the mechanisms underlying emotional reactions to music. The American Journal of Psychology.

[B36-behavsci-16-00761] Juslin P. N., Harmat L., Eerola T. (2014). What makes music emotionally significant? Exploring the underlying mechanisms. Psychology of Music.

[B37-behavsci-16-00761] Juslin P. N., Västfjäll D. (2008). Emotional responses to music: The need to consider underlying mechanisms. The Behavioral and Brain Sciences.

[B38-behavsci-16-00761] Kahn J. H., Enevold K. C., Feltner-Williams D., Ladd K. (2025). Using music to feel better: Are different emotion-regulation strategies truly distinct?. Psychology of Music.

[B39-behavsci-16-00761] Koelsch S. (2020). A coordinate-based meta-analysis of music-evoked emotions. NeuroImage.

[B40-behavsci-16-00761] Krause A. E., Dimmock J., Rebar A. L., Jackson B. (2021a). Music listening predicted improved life satisfaction in university students during early stages of the COVID-19 pandemic. Frontiers in Psychology.

[B41-behavsci-16-00761] Krause A. E., Glasser S., Osborne M. (2021b). Augmenting function with value: An exploration of reasons to engage and disengage from music listening. Music & Science.

[B42-behavsci-16-00761] Krause A. E., North A. C., Hewitt L. Y. (2015). Music-listening in everyday life: Devices and choice. Psychology of Music.

[B43-behavsci-16-00761] Krause A. E., Scott W. G., Flynn S., Foong B., Goh K., Wake S., Miller D., Garvey D. (2023). Listening to music to cope with everyday stressors. Musicae Scientiae.

[B44-behavsci-16-00761] Lamont A. (2011). University students’ strong experiences of music. Musicae Scientiae.

[B45-behavsci-16-00761] Liljeström S., Juslin P. N., Västfjäll D. (2013). Experimental evidence of the roles of music choice, social context, and listener personality in emotional reactions to music. Psychology of Music.

[B46-behavsci-16-00761] Lonsdale A. J., North A. C. (2011). Why do we listen to music? A uses and gratifications analysis. The British Journal of Psychology.

[B47-behavsci-16-00761] Lundqvist L., Carlsson F., Hilmersson P., Juslin P. N. (2009). Emotional responses to music: Experience, expression, and physiology. Psychology of Music.

[B48-behavsci-16-00761] McClymont R. G., Krause A. E. (2026). The impact of a focused listening experience on self-compassion and mental health help-seeking. Psychology of Music.

[B49-behavsci-16-00761] McKenzie S. M., Krause A. E., Glasser S., Osborne M. S. (2025). Exploring the role of music listening in cultivating self-compassion. Psychology of Music.

[B50-behavsci-16-00761] Memon M. A., Thurasamy R., Ting H., Cheah J.-H. (2025). Purposive sampling: A review and guidelines for quantitative research. Journal of Applied Structural Equation Modeling.

[B51-behavsci-16-00761] Muñiz J., Elosua P., Hambleton R. K. (2013). Guidelines for the translation and adaptation of the tests: Second edition. Psicothema.

[B52-behavsci-16-00761] Nawaz S., Omigie D. (2025). Qualities of music-evoked autobiographical memories are associated with auditory features of the memory-evoking music. PLoS ONE.

[B53-behavsci-16-00761] Nunnally J. C. (1978). Psychometric theory.

[B54-behavsci-16-00761] Ongchoco J. D. K., Melcher D. (2026). Aesthetic speed preferences when viewing dance synchronize to a ‘natural’ pace of human movement. Cognition.

[B55-behavsci-16-00761] Pas P., Hulshoff Pol H. E., Raemaekers M., Vink M. (2021). Self-regulation in the pre-adolescent brain. Developmental Cognitive Neuroscience.

[B56-behavsci-16-00761] Pekrun R. (2017). Emotion and achievement during adolescence. Child Development Perspectives.

[B57-behavsci-16-00761] Priyadarshini A., Divakarla U. (2026). RaagaDhvani: A novel augmented multi-feature dataset: Advancing emotion recognition in carnatic music with multimodal features and hybrid deep learning. Data in Brief.

[B58-behavsci-16-00761] Saarikallio S. (2007). Music as mood regulation in adolescence *(Jyväskylä Studies in Humanities, 67)*.

[B59-behavsci-16-00761] Saarikallio S. (2012). Development and validation of the brief music in mood regulation scale (B-MMR). Music Perception.

[B60-behavsci-16-00761] Saarikallio S., Erkkilä J. (2007). The role of music in adolescents’ mood regulation. Psychology of Music.

[B61-behavsci-16-00761] Saarikallio S., Vuoskoski J., Luck G. (2014). Adolescents’ expression and perception of emotion in music reflects their broader abilities of emotional communication. Psychology of Well-Being.

[B62-behavsci-16-00761] Saarikallio S. H., Maksimainen J. P., Randall W. M. (2019). Relaxed and connected: Insights into the emotional–motivational constituents of musical pleasure. Psychology of Music.

[B63-behavsci-16-00761] Saarikallio S. H., Randall W. M., Baltazar M. (2020). Music listening for supporting adolescents’ sense of agency in daily life. Frontiers in Psychology.

[B64-behavsci-16-00761] Sakka L. S., Juslin P. N. (2018). Emotion regulation with music in depressed and non-depressed individuals. Music & Science.

[B65-behavsci-16-00761] Schäfer K., Saarikallio S., Eerola T. (2020). Music may reduce loneliness and act as social surrogate for a friend: Evidence from an experimental listening study. Music & Science.

[B66-behavsci-16-00761] Schellenberg E. G., MacDonald R. A. R., Kreutz G., Mitchell L. (2012). Cognitive performance after listening to music: A review of the Mozart effect. Music, health, and wellbeing.

[B67-behavsci-16-00761] Schellenberg E. G., Mankarious M. (2012). Music training and emotion comprehension in childhood. Emotion.

[B68-behavsci-16-00761] Schermelleh-Engel K., Moosbrugger H., Müller H. (2003). Evaluating the fit of structural equation models: Tests of significance and descriptive goodness-of-fit measures. Methods of Psychological Research Online.

[B69-behavsci-16-00761] Schubert E. (2013). Loved music can make a listener feel negative emotions. Musicae Scientiae.

[B70-behavsci-16-00761] Tulving E. (1983). Elements of episodic memory.

[B71-behavsci-16-00761] Vandenberg R. J., Lance C. E. (2000). A review and synthesis of the measurement invariance literature: Suggestions, practices, and recommendations for organizational research. Organizational Research Methods.

[B72-behavsci-16-00761] Van Goethem A., Sloboda J. (2011). The functions of music for affect regulation. Musicae Scientiae.

[B73-behavsci-16-00761] Willimek B., Willimek D. (2022). Music and emotions—Research on the theory of musical equilibration (die Strebetendenz-Theorie). Eunomios.

[B74-behavsci-16-00761] Willimek D., Willimek B. (2011). Music und emotionen—Studien zur strebetedenz-theorie.

